# The effect of COVID-19 on the mental health of the people in the Indian subcontinent: A scoping review

**DOI:** 10.3126/nje.v13i2.52766

**Published:** 2023-08-31

**Authors:** Russell Kabir, Ancy Chandrababu Mercy Bai, Haniya Zehra Syed, Md. Rakibul Hasan, Divya Vinnakota, Sujita Kumar Kar, Rakesh Singh, Brijesh Sathian, S.M. Yasir Arafat

**Affiliations:** 1 School of Allied Health, Faculty of Health, Education, Medicine, and Social Care, Anglia Ruskin University, Chelmsford, United Kingdom; 2 Department of Public Health and Nursing, University of Sunderland London, UK; 3 Department of Psychiatry, King George's Medical University, Lucknow-226003, U.P., India; 4 Department of Research, Transcultural Psychosocial Organization Nepal (TPO Nepal), Kathmandu, Nepal; 5 Geriatrics and long term care department, Rumailah Hospital, Doha, Qatar; 6 Department of Psychiatry, Enam Medical College and Hospital, Dhaka-1340, Bangladesh

**Keywords:** Indian subcontinent, COVID-19, pandemic, Anxiety, Depression, Mental Health

## Abstract

Indian subcontinent has high mental heath burden and low resources to cope the mental health challenges. Assessment of impact of COVID-19 pandemic on the mental health would help to prioritize the resource allocations. We aimed to assess the impact of COVID-19 on the mental health of people in the Indian subcontinent. Following the PRISMA 2020 guideline, a scoping review was performed by systematically searching the PubMed, Scopus, and Embase databases to identify original studies that assessed mental health conditions during the COVID-19 pandemic in the Indian subcontinent. In this review, a total of 34 studies conducted between 2020 and 2022 were analyzed. The prevalence of anxiety disorders was found to range widely, from 2.5% in North Indian urban slum to 53% in Bangladesh and 21.7% in Pakistan. Similarly, the prevalence of depression varied widely, with rates ranging from 3.5% in North India to 29.8% in Pakistan. The prevalence of stress-related problems ranged from 18.3% in Pakistan to 59.7% in Bangladesh. Factors such as female gender, married status, healthcare workers, and mental illness were identified as important predictors of anxiety and depressive disorders. The impact of COVID-19 pandemic on mental health in Indian subcontinent varies widely based on study population and methods. Therefore, a cautious interpretation is needed while generalizing the study results.

## Introduction

The COVID-19 infection started in the Chinese city of Wuhan as a cluster of 40 pneumonia cases with no known cause. On January 30, 2020, the World Health Organisation (WHO) deemed the widespread viral outbreak a Public Health Emergency of International Concern.. The infection became widespread in a short time, with deaths reported throughout the globe. Following this, on 11th March, WHO declared COVID-19 as a global pandemic, reported by at least 114 countries [1]. The effect of COVID-19 was evident globally and at all levels of society, including economic, commercial, educational and psychological [2]. Besides the high morbidity and mortality (3-4%) of COVID-19, causing significant deaths worldwide, COVID-19 is also responsible for severe psychological deterioration among populations. The overwhelming spread of this respiratory virus led to global confusion, anxiety and fear, accompanied by hatred and stigma [3]. Social isolation, loneliness, general anxiety associated with health threats, and fear for themselves and their loved ones are some of the factors linked with worse physical and mental conditions. The psychological impact was worse on vulnerable groups like the elderly, children, immunocompromised individuals, and frontline workers [4]. Psychosocial co-morbidity is a shared public health burden among different countries and communities. People suffering from pre-existing psychiatric ailments such as depressive disorder, schizophrenia, obsessive-compulsive disorder (OCD) and dementia have exhibited exacerbations in response to stress diathesis, general fear of uncertainty, isolation, lacking access to resources for mental health, and reduced compliance and supervision [5,6]. COVID-19 has been found related to neuropsychiatric presentations in the form of delirium, anxiety disorders, depressive disorders, sleep problems and a higher incidence of self-harm [7].

Although COVID-19 is a global pandemic, it has affected each region differently. The impact of COVID-19 is particularly significant in low resource settings in developing countries with overwhelmed and fragile healthcare services. In the absence of an adequate workforce with non-existent resources and an unstable healthcare system, the general public suffers psychologically [8]. A systematic review was conducted to study the prevalence of depression and anxiety in South Asia during COVID-19. The prevalence of anxiety and depression overall, according to this survey, was 41.3% and 34.1%, respectively, with women showing higher trends for both disorders than their male counterparts. According to the study's findings, anxiety and depression are more prevalent in Bangladesh (52.3% and 48.2% respectively) and Pakistan (50.4% and 41.6%, respectively) than in other South Asian nations [9]. Most developing countries lack national-level mental health surveillance resources. These countries predominantly rely on local health workers, general physicians and independent researchers for mental health risk communication and community awareness during public health crises, such as COVID-19 pandemic [8, 10]. With a lack of resources to cope with the pandemic, mental health service is generally neglected in developing countries such as South Asia, despite increasing demand for these services [9].

There is documented evidence highlighting South Asia’s pre-pandemic mental health burden with limited access to resources. Our study aims to see the impact of COVID-19 on the mental health of people in the Indian subcontinent. The study seeks to understand the prevalence and progression of mental health disorders during the pandemic, thus providing essential data to understand the mental health situation. The study will pave the way for better decision-making in allocating mental health resources and policy making.

## Methodology

### Study Design

The goal of this study was to present a comprehensive picture of COVID-19's effects on mental health issues in the Indian subcontinent. It conducted a thorough search of the medical literature to find all relevant articles evaluating the state of people's mental health during the COVID-19 pandemic in the Indian subcontinent. It contained quantitative observational studies presenting original research evidence on this subject.

### Search Strategy

According to the Preferred Reporting Items for Systematic Reviews and Meta-Analyses (PRISMA) 2020 guidelines, a thorough search of the published literature was conducted. The Indian subcontinent, which is made up of the nations of Bangladesh, Bhutan, India, the Maldives, Nepal, Pakistan, and Sri Lanka, are the only countries that was searched for the relevant literature. To reduce bias and ensure important research was not overlooked, the search was carried out across multiple databases. The searched databases were PubMed, Embase, and Scopus electronically to obtain relevant research studies published from 1st January 2020 to 31st Jun 2022. To ensure that no eligible studies were missed, a second step involved manually checking the reference lists of currently eligible research.

The search strategy follows the SPIDER question framework and is helpful for mixed methods research topics focused on “samples” rather than populations.

**Figure 1.**
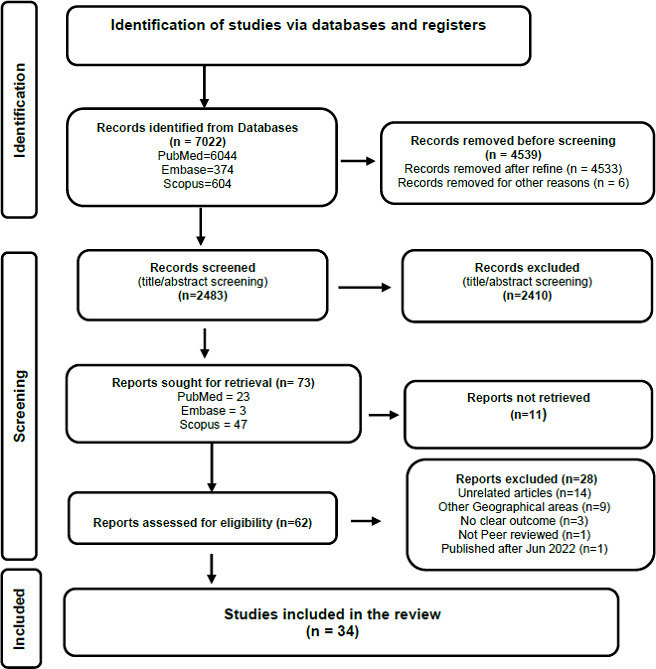
The PRISMA 2020 statement: an updated guideline for reporting systematic reviews

7022 articles were discovered through database searches, and 0 publications were discovered using reference harvesting (Figure 1). To limit the search results, boolean operators combinations, and truncations were used. The databases were searched using the following keywords: “COVID-19”, “Coronavirus” “Pandemic”, “Generalised anxiety disorder”, “Panic disorder”, “Social anxiety disorder”, “Separation anxiety disorder”, “Selective mutism”, “Specific phobia”, “Agoraphobia”, “Substance-induced anxiety”, “Anxiety disorder due to another medical condition”, “Bangladesh”, “Bhutan”, “India”, “Maldives”, “Nepal”, “Pakistan”, “Sri Lanka” either singly or in combination. The range of the search was reduced after the application of search restrictions to only primary, English-language, peer-reviewed papers and full-text articles.

### Study Selection process

Table 1 lists the inclusion and exclusion criteria:

The duplicated publications were removed using the RefWorks tool and manually examined to ensure there was no bias due to duplication before applying the inclusion and exclusion criteria. After the duplicate articles were eliminated, a total of 2483 publications were discovered in the literature search (Figure 1). Following title screening, 2410 records were eliminated. The retrieval of 73 reports was requested.

### Inclusion Criteria and Exclusion Criteria Implementation

The first phase involved screening the papers discovered as a result of the search for research design. Then, “SPIDER” inclusion and exclusion criteria for sample and study designs that were restricted to interventional and non-interventional studies and observational studies were applied for the screening of title and abstract of each search result. In the next screening phase, 73 relevant articles selected on the effect of COVID-19 (P- Phenomenon of Interest) in the Indian Subcontinent (S-Sample) resulted from the search. Their full-text articles were scanned for data collected from participants who had an anxiety disorder or not (E-Evaluation) and searched for the outcome. The literature search found that in some articles, the population group was a global population; in some, it was a mixed population of the Indian subcontinent and other countries. These articles were excluded during this stage. Also removed were any articles that provided insufficient or unfocused information about the anxiety disorder. 34 publications were chosen for the critical appraisal stage after the inclusion and exclusion criteria were implemented (Figure 1 and Table 1).

### Data Extraction

Microsoft Excel was used to extract the data. Data regarding the article's in-text citation, the study's design, its context (such as "region, country"), sample size, whether anxiety disorders were the study's primary focus, its goal, results, and study’s limitations were extracted.

### Analysis

The extracted data from the studies were organised, and examined, followed by a textual narrative synthesis.

### Ethical aspects

We reviewed secondary information from publications with open access. As a result, no approval from the institutional review board was sought to carry out the study.

## Results

### Characteristics of the included studies

34 papers in total were taken into consideration for this study based on the inclusion criteria (Table 3). Since the publications were chosen based on the Covid-19 pandemic timeframe, all of the research was carried out between 2020 and 2022. The most often used scales for identifying anxiety and deprssive disorders were Patient Health Questionnaire (PHQ-9) [21, 28, 33, 35, 39, 41], Generalized Anxiety Disorder (GAD-7) ) [11, 14, 19, 21, 25, 27-29, 33, 39, 41], Hospital Anxiety and Depression Scale (HADS-14) [24], and Depression Anxiety Stress Scales (DASS-21) [18, 30, 32, 34]. Most research looked at anxiety, depression, and sleep quality. At the same time, rest focussed on mental health issues, fear, stress, panic and other psychological issues. Among 34 studies, twelve studies were published from India, ten from Bangladesh, six from Nepal, five from Pakistan, and one study was published from Bhutan. No study was identified from Maldives and Sri Lanka. The studies used a variety of samples with varying age groups, socioeconomic positions, and educational levels. Sample size ranged from 70 in Pakistan [20] to 3122 in Bangladesh [18]. Six studies were conducted among community populations, nine studies were conducted among students, two studies conducted among slum populations, four studies were conducted among peri-partum period, six studies were conducted among health care providers, and three studies were conducted among COVID-19 patients.

In India, twelve studies assessed anxiety, six studies depression, one study stress and two studies sleep disturbances. In Bangladesh, anxiety was identified in nine articles, seven articles of depression and one each in stress and sleep quality. Both Nepal and Pakistan have five studies that assessed anxiety whereas only one in Bhutan. Two studies in Nepal were found on depression, four in Pakistan and one in Bhutan. Both Nepal and Pakistan have one article on stress.

### Design of studies

Among the selected 34 papers, 33 were cross-sectional studies and one was a cohort study. The studies employed data collection methods such as internet-based surveys, phone based surveys, face to face interviews and online surveys.

### Anxiety

The prevalence of anxiety among various population groups ranges from 2.5% to 57.2% [11, 25, 31, 35, 36]. Around 31% of front-line healthcare workers (HCWs) reported anxiety [24]. In Bangladesh, residents reported mild to moderate anxiety [25] whereas in Pakistan severe and extremely severe anxiety was reported [36] and medical health workers (doctors, nurses, and allied healthcare professionals) reported borderline to abnormal anxiety (37.17%) [24]. There was a statistically significant correlation between working in a primary or secondary level healthcare facility and having higher levels of anxiety among surgeons [37]. Nurses were discovered to be twice as likely to have anxiety symptoms as physicians. Similar to nurses, laboratory workers had roughly three times the likelihood of anxiousness. HCWs who lived in a combined family are two times more likely to display signs of anxiety [30]. A significant predictor of probable GAD was discovered to be the presence of high-risk family members [29]. Females are more likely to have anxiety in comparison to males [11, 12, 17, 19, 30, 44] and single people are less likely to be anxious when compared to married people [11, 17, 37, 44]. Shared facilities, food availability, food purchasing ability, fear of contracting COVID-19, and concern over family members contracting the disease were all strongly linked to anxiety [25]. The risk of the corona virus infecting the foetus (40.6%), the risk of the virus infecting the mother owing to pregnancy, and the risk of the virus infecting the mother during labour (37.7%) were the most often stated concerns of anxiety among pregnant women [40]. Additional anxiety predictors included a history of mental illness and the importance of a life event, whereas any family member dying from Covid-19, the magnitude of the event, and the length of the quarantine enhanced anxiety [42]. In patients who regularly receive Covid-19 crucial updates, the prevalence of GAD has been found to be considerably greater. Around 73.2% of the concerned respondents feared getting the COVID-19 infection [44]. Comorbidity and the presence of at least three COVID-19 physical symptoms were linked to anxiety symptoms. Additionally, anxiety among students was marginally connected with concerns about academic delays [14].

### Depression

The prevalence of depression among residents of North India is reported to be 3.5% [35]; whereas in Pakistan 29.8% of severe and extremely severe cases of depression were identified [36]. Among geriatric population, 18.87% had depressive symptoms during covid-19 [26] whereas females (64.3%), those under the age of 23 (62.8%), and the unemployed (77%) also had a higher prevalence of depressed symptoms [12]. Similarly, medical health workers (doctors, nurses, and allied healthcare professionals) (19.12%), front-line healthcare workers (17.6%) [24]. Married respondents were found to be strongly associated with depression [36]. According to the results of one study, depression is predicted by life occurrences and a history of psychiatric illness [42]. While HCWs working in high-risk environments had almost a two-fold increased risk of developing depressive symptoms, HCWs living in nuclear households with insufficient or no personal protective equipment (PPE) had almost a three-fold increased risk [30]. Additionally, the existence of comorbidity, having three or more Covid-19 physical symptoms, and having an oxygen saturation below 93% were all linked to depressed symptoms [31].

### Stress

According to the study findings from Pakistan, 18.3% of respondents were identified with stress and females are strongly associated with stress [36]; 59.7% suffered from symptoms of stress [12]. Medical health workers (doctors, nurses, and allied healthcare professionals) reported moderate to high stress levels (66.66%) [24]. Nearly two thirds (62.4%) of front-line healthcare workers reported experiencing moderate to severe stress [23]. Significant correlations were found between higher levels of stress and unemployment (71.9%), females (70.9%), and monthly income (63.8%) in Bangladesh [12].

### Stress, Anxiety and Depression

Among North Indians, during COVID-19 pandemic around 4% of them either had depression or anxiety and 2% reported to have both [35]. There are greater instances of stress, anxiety, and depression among respondents who worry about new COVID-19 cases and deaths and those who spend more than 6 hours on social media. Additionally, those who think they have a better probability of survival are less likely to experience sadness, anxiety, and stress [36]. Adults in Bangladesh experienced higher levels of anxiety and depression throughout the COVID-19 period for a number of reasons, including increasing worry about themselves and the family members' futures and health-related characteristics [17]. Stress levels, anxiety symptoms, and depression symptoms were all substantially correlated with marital status and occupation in Bangladesh [12]. For depression and anxiety, those who are academically behind are more inclined to self-report mental health symptoms than people who are not [41].

### Insomnia

Residents from Bangladesh states 43% had mild to moderate insomnia [25]. Significant correlations were found between insomnia and sex, shared spaces, the ability to buy food, COVID-19 fear, family member COVID-19 fear, and having someone like friends or neighbours have COVID-19 [25]. Working in primary or secondary healthcare facilities was potentially related to sleep disturbance, although age, educational level, the availability of PPE, and Covid-19 training were not significantly related to sleep pattern [37]. Significant predictors of low sleep quality included the existence of addictive behaviours, unprotected interaction with COVID-19 individuals, and suspected generalised anxiety disorder [29]. Sleep quality disturbance was found to be statistically significant with severity of illness as well [43].

## Discussion

The prevalence of anxiety and depression varies among population groups and countries. A study from North India links the pandemic to increased mental health disorders [45]. This study was conducted among migrant workers who lost their jobs and residences due to the epidemic, resulting in increased anxiety and depression.

Several research conducted during the COVID-19 pandemic revealed that governmental isolation has a detrimental effect on older people's mental health, especially when it results in a decline in wellbeing, a lack of social activities, and an increase in loneliness [46-49]. In the current study, findings from various studies identified that during this pandemic, sleeplessness, anxiety and depression was considerably more common in women than in men. Numerous studies conducted during this pandemic also shown that women were more likely than males to have mental health symptoms such anxiety and insomnia [50-52]. This gender discrepancy in mental illness is caused by a number of biological, hormonal, social, and cultural factors [53, 54].

A HCWs’ own safety and the welfare of his or her family are two significant sources of stress. This was clear from the study's findings, which showed that married surgeons had higher levels of anxiety than single surgeons [37]. According to a survey conducted by the *University of Arkansas for Medical Sciences* to assess and assure the well-being of their physicians, the safety of their family in this pandemic is the top concern for all HCWs [55]. According to another study, nurses and lab workers were more likely than doctors to experience feelings of anxiety among HCWs and among females [36]. Being female and working as a nurse were linked to anxiety symptoms in a study of HCWs carried out in Nepal and India during the COVID-19 epidemic [56,57]. Nurses spend a lot more time caring for patients than other healthcare professionals do. However, laboratory workers may experience anxiety symptoms due to the volume of work they must do and the roles they play in diagnosing COVID-19.

Almost half of the population believes that the COVID-19 outbreak has given rise to unwarranted rhetoric. In times of crisis, people are prone to looking for crucial information. Lack of access to reliable information can make people more anxious [58].

Many women are given misleading information about COVID-19 and breastfeeding, which can lead to mothers being prevented from nursing or children being taken away. During the early stages of the epidemic, mothers and newborns were often separated, and breast milk replacements were sometimes prescribed [59]. Some women stopped nursing out of concern that COVID-19 might spread, while others struggled with depression and anxiety about their child's health [15].

Studies around the world have found that college students experience depression and anxiety due to COVID-19 related stressors such as financial burdens and academic delays. Chinese students' anxiety is linked to fear of activity delays [60]. However, another study noted that while the switch to online classes was challenging for them, the students' top concern (82%) was related to their academic performance [61].

## Strengths and limitations of the study

This paper is the first scoping review on the effect of Covid-19 pandemic on the mental health of people in the Indian Subcontinent, to the best of the authors' knowledge. However, it should be noted that the study's focus on peer-reviewed literature may have excluded relevant articles published in local journals. Furthermore, the review's inclusion of only English-language studies introduces geographical and linguistic biases. Lastly, excluding articles that are not available in full text may have resulted in the omission of recent research findings.

## Conclusion

This review assessed the presence of anxiety, depression, stress and sleep disturbances caused by the pandemic and its impact on people’s health in the Indian Subcontient. The findings of this review identified that females, especially married women, HCWs, students, general population with medical conditions and pregnant women are mostly affected, multiple factors contributed to the issues. It was clearly shown the focus on mental health issues is important for psychological well-being and good quality of life. The current findings will aid direct efforts to develop policies that will give healthcare providers the tools they need to address mental health issues among various demographic groups, especially when more severe symptoms might be present during crises like the COVID-19 pandemic.

## Figures and Tables

**Table 1:** Inclusion and Exclusion Criteria

	Inclusion Criteria	Exclusion Criteria
**S**ample	Indian Subcontinent (Bangladesh, Bhutan, India, Maldives, Pakistan and Sri Lanka) population	Other geographical area populations other than the Indian subcontinent
**P**henomenon of **I**nterest	Coronavirus Disease (Covid-19)	Articles did not collect data on Coronavirus disease
**D**esign	Interview, questionnaire, survey (online and individual)	Articles did not collect and evaluate data based on established questionnaires or methods
**E**valuation	Rate of Mental Health Conditions : Anxiety disorder Depression Stress disorders Sleep problem	Articles did not collect data regarding the Mental Health Conditions
**R**esearch type	Interventional studies Observational Studies Articles in the English Language Literature completed after 2011	Systematic review and meta-analyses Case studies with less than five subjects Articles written in languages other than English Literature completed prior to Jan 2020 and after Jun 2022

**Table 2:** Study details

Sl. No	Study	Domain assessed	Study Setting (Location)	Sample Size	Study Design	Measuring Instruments	Key Findings
**1.**	Ali et al., 2022 [11]	Anxiety	General Public of Karachi	331	Cross-Sectional Study	GAD-7 scale	48.9% of the participants reported anxiety and related disorders. It revealed no significant relationship of anxiety disorders with preventative behaviours. However, a significant association was reported between anxiety-related disorders and sociodemograhics.
**2.**	Banna et al., 2022 [12]	Mental health among a home-quarantined population	Bangladesh	1,427	Prospective Cross-sectional Web-based Study design	Utilising WHO materials on the COVID-19 Pandemic, an online survey questionnaire was created. Three sections made up the survey: Participant characteristics Perceptions regarding COVID-19 Mental health	The study reported a significantly higher level of mental health symptoms in Bangladesh during the COVID-19 pandemic. The study also reports lower pre-COVID levels of mental health conditions amongst adult population of Bangladesh (6.5% - 31%), attributing the increasing levels of these issues to the pandemic. However, the study is prone to sampling bias due to lack of access to internet among the population alongwith gender and occupational preference.
**3.**	Banstola et al., 2021 [13]	Anxiety	A tertiary hospital in Nepal	144 nursing students	Descriptive Cross-sectional study	A self-administered online questionnaire with 10 items that included demographic, clinical, and COVID-19-related questions was used. 21 questions in the Anxiety Inventory are used to evaluate the symptoms of anxiety and 28-item Brief-COPE to evaluate the strategies for coping.	The paper reported anxiety amongst all the nursing students during clinical placements and subsequently mentioned various coping strategies used to overcome anxiety, most popular of which was ‘Religion’. However, due to self-reporting nature of the survey, it is prone to response bias and due to a limited sample size, generalizability cannot be established.
**4.**	Dangal et al., 2020 [14]	Anxiety	Nepal	105 Students	Quantitative Cross-sectional descriptive study	GAD-7 scale	Amongst the participants who reported to have anxiety, 25.7% reported mild anxiety, 22.9% experienced moderate anxiety and 18.1% experienced severe anxiety. Increased anxiety was reported amongst female gender.
**5.**	Doshi et al., 2021 [15]	Anxiety, fear and depression	Lokmanya Tilak Municipal Medical College and General Hospital, Sion, Mumbai, India	A total of 62 COVID-positive postpartum mothers	Prospective Cross-Sectional Observational	corona disease anxiety scale (CDAS), FCV-19S, and Edinburgh postpartum depression scale (EDPS).	Moms who tested positive for COVID after giving birth experienced an increase in their mental health issues, such as anxiety and mood problems.
**6.**	Faisal et al., 2022 [16]	Anxiety, depressive symptoms, and mental health status	Bangladesh	874 Bangladeshi students attending universities either in Bangladesh or abroad	Quantitative Study	GAD-7, the Mental Health Inventory-5 and the Depression Revised Scale.	The findings of this study showed that university students in Bangladesh suffered substantial levels of psychological distress. These results might shed light on how the pandemic's uncertainty is adversely affecting university students in Bangladesh now that they are back in a virtual or hybrid learning environment.
**7.**	Haque et al., 2022 [17]	Anxiety and depression	Bangladesh	460 Participants aged 18	Cross-Sectional Survey	a 12-item measure for anxiety; a 12-item scale for depression.	The study found a significant correlation between anxiety and depression scores during COVID-19 lockdown and respondents' concerns for their future well-being, perceived physical health status compared with others in the same cohort, and perceived physical health status compared to prior COVID-19.
**8.**	Islam et al., 2020 [18]	Depression, anxiety and stress	Bangladesh	3,122 university students aged 18 to 29 years	Cross-Sectional Design	DASS-21	The study reported 70-76% respondents experiencing mild symptoms of depression, anxiety and stress. 19-28% of the respondents reported very severe symptoms of depression, anxiety and stress. The study also reports a higher score of DASS-21 amongst Bangladeshi university students than the pre-COVID-19 scores.
**9.**	Islam et al., 2020 [19]	Panic and anxiety	Bangladesh.	1311 community dwelling individuals aged between 13 and 63 years	Cross-Sectional Online Survey	GAD-7 and the Panic Disorder Severity Scale	Panic was estimated at 79.6% and general anxiety at 37.3%. Statistically significant predictors of panic included being over 30, having higher education, being married, and having a large family. Being a woman, being older (more than 30 years), having more education (than a bachelor's degree), being married, and working for an organisation other than the government were all factors that statistically predicted generalised anxiety.
**10.**	Jafri et al., 2022 [20]	Anxiety and Depression	Lahore, Pakistan	70 individuals (either gender) between the age of 18–60 years, who contracted COVID-19 previously and then recovered as indicated by negative PCR	Cross-Sectional Study	impact of event scale (IES-R), PHQ-9 and corona anxiety scale (CAS)	32.9% of these participants reported no symptoms, 67% demonstrated common COVID-19 symptoms and 75.7% experienced post-recovery symptoms of COVID-19. While 74.3% of partcipants with COVID-19 symptoms reported no anxiety (as assessed from the Corona Anxiety Scale), post-traumatic stress was common among respondents especially those who were symptomatic. Mild depression was also reported among the respondents who had COVID-19 symptoms.
**11.**	Jain et al., 2021 [21]	Anxiety disorder, mood disorders and stress	Medical and engineering colleges in Bihar, Delhi and Maharashtra, and Tamil Nadu. (India)	699	Cross-Sectional Multicentric Study	Corona virus anxiety screening (CAS), the General Health Questionnaire (GHQ)-12, GAD-7 scale, and PHQ-9	The study reports that when assessed with CAS, majority of the respondents did not cross the threshold score. However, for GHQ-12 majority of the participants scored above the threshold score (62-64%). Both the groups of students i.e. MBBS and Engineering students reported mild anxiety and mild depressive symptoms with insignificant differences between the two groups. Academic delays and uncertainty about exams alongwith fear of vulnerability to COVID and isolation were reported to be major stressors for these students.
**12.**	Jeelani et al., 2022 [22]	Depression and anxiety	Kashmir valley of India	426 school-going adolescents aged between 15 and 19 years	Cross-Sectional Study Design	GAD-7 and PHQ-9.	The study reports anxiety in 20% of adolescents and depression among 16% of the respondents with an association to previous COVID-19 infection. The study also reports higher prevalence among females (27.5%) as compared to their male counterparts (14%).
**13.**	Kafle et al., 2021 [23]	Psychological distress	Nepal	254 health service providers	Web-Based Cross-Sectional Survey	COVID-19 Peritraumatic Distress Index (CPDI) questionnaire	The majority of participants—46.9% were not distressed, while 46.5% were mild to highly distressed (score >28 to 51), and 6.7% were extremely distressed (score 52). Participants who were doctors by profession (p = 0.001) and those who were female (p = 0.004) reported considerably higher levels of distress.
**14.**	Kafle et al., 2021 [24]	Depression, anxiety and stress	A teritary centre of Nepal	280 health care workers	Hospital based descriptive cross-sectional study	HADS-14,Perceived stress scale.	Approximately 36% of the healthworkers worked at the frontline during the pandemic. Of the total repondents, prevalence of stress was most common (74%) followed by anxiety (41.4%) and depression (24.1%).
**15.**	Koly et al., 2021 [25]	Anxiety and insomnia	Dhaka city, Bangladesh	586 adult residents of five informal settlements	Cross-Sectional Phone-Based Survey	GAD-7 and the Insomnia Severity Index (ISI).	Mild to severe anxiety and sleeplessness were both present with a prevalence of 53% and 43%, respectively. Participants who experienced mild anxiety were considerably more likely to be older (>50 years old) and concerned about contracting the COVID-19 virus. Participants with moderate to severe anxiety were also significantly more likely to share fewer household amenities (such as the toilet, kitchen, and water) (OR: 2.23; 95% CI: 1.31-3.79), experience problems getting enough food (OR: 2.76; 95% CI: 1.10-6.93), be afraid of themselves (OR: 5.27; 95% CI: 2.82-9.88), and worry about infecting family members (OR: 2.26; 95% CI: 1.23-4.17).
**16.**	Kumar et al., 2021 [26]	Mental health problems	Tertiary health care facility of India	106 participants (Age ≥60 years) of either gender	Cross-sectional Observational study	Hamilton Anxiety Rating Scale (HAM-A) and Geriatric depression scale (GDS)	Twenty patients (18.87%) on GDS and twenty-four (22.6%) patients on the HAM-A reported having depression and anxious symptoms, respectively. It is important to remember that the study shows that the geriatric population experienced severe mental health problems during the COVID-19 epidemic.
**17.**	Marbaniang et al., 2020 [27]	Anxiety	Pune, India.	167 People Living with HIV (PLHIV)	48-month Prospective Cohort Study	GAD-7	The Generalised anxiety was 25% more common overall (n = 41). Less ART dosages were still needed for PLHIV with a GAD-7 score of 10 or above (p = 0.05).
**18.**	Mehareen et al., 2021 [28]	Depression, and anxiety	Two public and three private universities in Bangladesh	333	Cross-Sectional Study	PHQ-9 and GAD-7	The study reports significant association of female gender, lower socio-economic status, nuclear family and advance study levels with a higher score of depression, anxiety and related co-morbidities.
**19.**	Naik et al., 2022 [29]	Anxiety and sleep quality	All India Institute of Medical Sciences, Patna	370 Healthcare Professionals	Cross-Sectional Study	PSQI and GAD-7	52 (14.1%) of the 370 healthcare professionals examined had GAD, and 195 (52.7) had poor sleep. There was a substantial percentage of healthcare professionals who reported having trouble sleeping and suspected GAD.
**20.**	Pandey et al., 2021 [30]	Stress, anxiety, depression and their associated factors	Nepal	404 Health Care Workers	Web-Based Cross-Sectional Survey	DASS-21	The study reports approximately 36% prevalence of anxiety among healthcare workers. The study shows that females are two times more likely to have symptoms of anxiety and depression than their male counterparts. Moreover, laboratory workers are reported to be three fold more susceptible and nurses twice more susceptible to anxiety, when compared to doctors. Healthcare workers with no/limited access to PPE were reported to be three times more vulnerable to depression.
**21.**	Rahman et al., 2021 [31]	Psychological conditions during this period.	Bangladesh	1,382 university students	Cross-Sectional Study	DASS-21	Approximately 27% students experiencing anxiety and approximately 25-29% reporting mild to moderate depression. The study reports factors such as age, academic level, area of academia, safe living arrangements, academic performance, social life and job security to be strongly associated with symptoms of anxiety and depression.
**22.**	Rahman et al., 2021 [32]	Anxiety and depressive symptoms	Three isolation facilities in Dhaka, Bangladesh	138 COVID-19 patients	Cross-Sectional Study	The *Hospital Anxiety and Depression Scale (HADS)* was used.	Almost half of the patients had symptoms of anxiety (57.2%) and depression (52.2%). Moreover, the patients with co-morbidities and exhibiting 3 or more physical symptoms associated with COVID-19, reported symptoms of anxiety.
**23.**	Ray et al., 2022 [33]	Anxiety & depression	KPC Medical College, Kolkata, India	124 patients	Cross-Sectional Study	GAD-7 and PHQ-9	31 (25%), 36 (29.03%), and 21 (16.94%) participants, respectively, were diagnosed with anxiety, depression, and both anxiety and depression. Fears about COVID-19's possible risks to mother and child's lives, fears about missing out on essential medical and obstetric treatment during the lockdown, social isolation, and unemployment during a pandemic were all linked to higher levels of anxiety and depression symptoms.
**24.**	Reddy et al., 2020 [34]	Anxiety	Indore, Madhya Pradesh, India	247	Descriptive, Cross-Sectional Study	DASS-21	The results showed that participants' ages and anxiety levels were statistically significantly correlated (p=0.021), with low anxiety scores (92.7%). The main drawback was the small sample size and uneven distribution of the study samples among the various categories as a result of the strict inclusion criteria for participants with English language comprehension.
**25.**	Rehman et al., 2021 [35]	Depression and anxiety.	The service area of a Public Health Dispensary (PHD), Chandigarh, India	200	Descriptive Cross-Sectional Study	PHQ-9 and GAD-7	3.5% (95% [CI]: 0.95-6.05) and 2.5% (95% CI: 0.34-4.66) of people reported having depression or anxiety, respectively. In total, 46.5% (n = 92) were aware that COVID-19 can spread by droplets, and 30.5% (n = 61) were worried they might contract the illness. 78% of participants (n = 156) covered their mouths when they coughed or sneezed, and 50% of participants (n = 100) said COVID-19 was not a cause for unnecessary concern.
**26.**	Shahid et al., 2022 [36]	Depression, anxiety and stress.	Karachi, Pakistan	2,069	Cross-Sectional Survey	(1) Generalized Questionnaire and (2) Mental health Questionnaire.	This study's findings show that 27.8, 21.7, and 18.3% of respondents, respectively, have severe and extremely severe states of depression, anxiety, and stress. An extremely severe state of depression, anxiety, and stress affects those who think the COVID-19 pandemic has negatively affected them psychologically, fear additional COVID-19 cases and deaths, are depressed as a result of the lockdown.
**27.**	Sharma et al., 2021 [37]	Anxiety and sleep	Orthopaedic surgeons practising in India	100 male orthopaedic surgeon	Online Cross-Sectional Study	GAD-7 scale, single-item sleep quality scale	8% of surgeons had severe anxiety, 12% had moderate anxiety, 27% had mild anxiety, and 53% had minor anxiety. 65% of respondents acknowledged that the epidemic has changed their management practises. Additionally, it discovered a connection between primary or secondary healthcare facilities and higher levels of anxiety in surgeons (p = 0.04).
**28.**	Shrestha et al., 2021 [38]	Anxiety	Kathmandu Medical College and Teaching Hospital, Nepal	273	Descriptive Cross-Sectional Study	*Hamilton Anxiety Rating Scale (HAM-A)*.	Only 2 (0.73%) of the 273 total patients had a score between 25 and 30, indicating moderate to severe anxiety, 21 (7.69%) had a score between 18 and 24, indicating mild to moderate anxiety, and 250 (91.57%) had a score between 0 and 17, indicating mild state.
**29.**	Tasmin et al., 2021 [39]	Depression and anxiety	Bangladesh	971	Cross-Sectional Study	The PHQ-9 and GAD-7	Anxiety and depression estimates ranged from moderate to severe, with 35.2% and 38.9%, respectively. People with medical disorders are more likely to experience depression and anxiety, and these feelings are correlated with sociodemographic, quality-of-life, and smoking variables.
**30.**	Tikka et al., 2021 [40]	Anxiety	Antenatal clinics of five medical college hospital in India	620 pregnant women	Cross-sectional study	The GAD-7 and a customized scale to assess antenatal COVID-19 anxiety	According to GAD 7, 11.1% of women had moderate or severe anxiety. Higher perception of the COVID-19 risk, more antenatal COVID-19 anxiety, and lower perceived support all strongly predicted moderate and severe generalised anxiety.
**31.**	Tshering et al., 2022 [41]	Depression and anxiety level	College of Science and Technology (CST), Phuentsholing, Bhutan	278 students	Web-Based Cross-Sectional Study	PHQ-9 and GAD-7	Approximately 70% of the respondents were male. The study reports approximately 28% anxiety and 44% moderate to severe depression among these students. Moreover, the students with family members working in frontline jobs reported a significantly greater score of anxiety.
**32.**	Um-e-kalsoom et al., 2022 [42]	Depression and anxiety	Community of Peshawar, Khyber Pakhtunkhwa (Pakistan),	320	Cross-Sectional Study Design	*Hospital Anxiety and Depression Scale*, Impact of *Life Event Scale-Revised*, and *Center of Epidemiologic Studies-Depression Scale*	According to this study, people affected by COVID-19 (47%), score significantly high on the depression and anxiety scales. Regression shows an association between history of psychiatric illness, duration of quarantine and impact of life events, and risk of depression. Similar factors have been reported to be associated with the risk of anxiety.
**33.**	Yadav et al., 2021 [43]	Depression, anxiety, and sleep disturbance.	COVID-19 hospital of a tertiary care centre in rural Uttar Pradesh, north India	100 COVID-19 patients	Hospital-Based, Cross-Sectional Study	PHQ-9 scale, GAD-7 scale and *PSQI*.	According to this study, 67% of patients had anxiety, 27% of patients had depression, and 62% of patients had sleep problems. With regard to comorbidity and illness severity, depression and anxiety were shown to be substantially correlated (p 0.05). A statistically significant relationship between sleep disturbance and illness severity was also discovered (p 0.05).
**34.**	Yasmin et al., 2021 [44]	GAD and depressive symptoms	Four provinces of Pakistan	500 participants who had access to the Internet and understood Urdu were included in the survey.	An Online Web-Based Cross-Sectional Survey	GAD-7 scale and the *Center for Epidemiology Scale for Depression (CES-D)* scale.	The survey's findings showed that 25,4% of respondents had GAD and 18,8% had depressive symptoms. Additionally, around 34.8% of participants expressed a fear of contracting COVID-19, 62.8% often received negative updates about COVID-19, and 17.6% could not comprehend the information on COVID-19. GAD was found to be substantially correlated with gender, age, and checking often critical updates for COVID-19.

GAD-Generalized Anxiety Disorder; FCV-19S- fear of COVID-19 scale; DASS- Depression, Anxiety, Stress Scale; PHQ-patient health questionnaire ; PSQI-Pittsburgh Sleep Quality Index
